# The Important Roles of Natural Killer Cells in Liver Fibrosis

**DOI:** 10.3390/biomedicines11051391

**Published:** 2023-05-08

**Authors:** Ming Yang, Ethan Vanderwert, Eric T. Kimchi, Kevin F. Staveley-O’Carroll, Guangfu Li

**Affiliations:** 1 Department of Surgery, University of Missouri, Columbia, MO 65212, USA; yangmin@health.missouri.edu (M.Y.);; 2 NextGen Precision Health Institute, University of Missouri, Columbia, MO 65212, USA; 3 Harry S. Truman Memorial VA Hospital, Columbia, MO 65201, USA; 4 Department of Molecular Microbiology and Immunology, University of Missouri-Columbia, Columbia, MO 65212, USA

**Keywords:** liver fibrosis, hepatic stellate cells, natural killer cells, cellular and molecular mechanisms, treatments

## Abstract

Liver fibrosis accompanies the development of various chronic liver diseases and promotes their progression. It is characterized by the abnormal accumulation of extracellular matrix proteins (ECM) and impaired ECM degradation. Activated hepatic stellate cells (HSCs) are the major cellular source of ECM-producing myofibroblasts. If liver fibrosis is uncontrolled, it may lead to cirrhosis and even liver cancer, primarily hepatocellular carcinoma (HCC). Natural killer (NK) cells are a key component of innate immunity and have miscellaneous roles in liver health and disease. Accumulating evidence shows that NK cells play dual roles in the development and progression of liver fibrosis, including profibrotic and anti-fibrotic functions. Regulating NK cells can suppress the activation of HSCs and improve their cytotoxicity against activated HSCs or myofibroblasts to reverse liver fibrosis. Cells such as regulatory T cells (Tregs) and molecules such as prostaglandin E receptor 3 (EP_3_) can regulate the cytotoxic function of NK cells. In addition, treatments such as alcohol dehydrogenase 3 (ADH3) inhibitors, microRNAs, natural killer group 2, member D (NKG2D) activators, and natural products can enhance NK cell function to inhibit liver fibrosis. In this review, we summarized the cellular and molecular factors that affect the interaction of NK cells with HSCs, as well as the treatments that regulate NK cell function against liver fibrosis. Despite a lot of information about NK cells and their interaction with HSCs, our current knowledge is still insufficient to explain the complex crosstalk between these cells and hepatocytes, liver sinusoidal endothelial cells, Kupffer cells, B cells, and T cells, as well as thrombocytes, regarding the development and progression of liver fibrosis.

## 1. Introduction

Liver fibrosis is associated with the progression of various chronic liver diseases, such as hepatitis viral infection and alcoholic or non-alcoholic steatohepatitis (ASH or NASH). It is characterized by the abnormal accumulation of extracellular matrix proteins (ECM, e.g., collagen and integrin) and impaired ECM degradation (e.g., metalloproteinases) [1]. Hepatic stellate cells (HSCs), the vitamin A-storing cells in the healthy liver, can be activated and differentiated to ECM-producing myofibroblasts in chronic liver diseases, leading to liver fibrosis [2,3]. Without effective treatments, liver fibrosis can progress to cirrhosis, causing liver cancer or failure. Unfortunately, we still lack approved treatments for liver fibrosis. Current strategies for liver fibrosis treatment mainly are limited to the protection and prevention of liver injury and fibrosis-causing factors. These strategies include treatments of anti-etiology (e.g., anti-viral treatment and reduction in alcohol consumption), anti-inflammation, antioxidant stress, inhibition of cell apoptosis, genetic and epigenetic modification, inhibition of HSC activation and proliferation, and promotion of ECM degradation [4,5,6]. Thus, exploration of new treatment options for liver fibrosis is urgently needed.

In the liver, both innate and adaptive immune cells play a pivotal role in the development of liver fibrosis and its associated liver diseases [7], such as alcoholic liver disease (ALD) [8,9], non-alcoholic fatty liver disease (NAFLD) [10,11], and liver cancer [12,13]. Natural killer (NK) cells are an essential component of innate immunity. In humans, NK cells comprise approximately 5–15% of circulating lymphocytes [14]. About half of the intrahepatic lymphocytes (50%) are NK cells, and they modulate the intrahepatic immune response in both physiological and pathological conditions [15]. In laboratory inbred mice, NK cells constitute about 2–5% of lymphocytes in spleens and bone marrows, and the number of NK cells doubles in wild-type mice [16]. In mouse livers, NK cells comprise about 2–5% of non-parenchymal cells (NPCs), and the frequency can be increased to around 10% in NASH livers of mice treated with high-fat and high-sugar diets [17,18].

NK cells have miscellaneous roles in liver health and disease [19,20]. For example, NK cells can be recruited by C-X-C motif chemokine ligand 9 (CXCL9), produced in activated CD103^+^DCs (dendritic cells), into the tumor microenvironment to express granzyme B, interferon-gamma (IFN-γ), and tumor necrosis factor-alpha (TNF-α) to kill HCC cells [21]. NK cells also have anti-viral infection capabilities. A study showed that there was a negative correlation between the deoxyribonucleic acid (DNA) titer of hepatitis B viruses and the frequency of CD56^bright^NK cells in CHB patients with chronic hepatitis B (CHB) and peginterferon alpha-2a treatment [22]. In addition, the expression of interferon alpha receptor 2 (IFNAR2) and NK cell p46-related protein (NKp46) in NK cells was remarkably increased in patients with functional cure compared to that in CHB patients without functional cure. Furthermore, NK cells are implicated in the pathogenesis of liver fibrosis. The frequency of peripheral blood NK cells (CD3^−^CD16^+^CD56^+^) was increased in patients with alcoholic liver fibrosis (ALF) compared to that in patients without ALF. NK cell frequency was negatively correlated with total T cell frequency but positively associated with the percentage of CD3^−^CD8^+^ cells [23]. In the murine NASH model, DX5^+^ (anti-CD49b antibody) NKp46^+^NK cells can prevent liver fibrosis progression by suppressing M2-like macrophage polarization and the expression of profibrotic genes (e.g., *Tgfb1* encoding transforming growth factor-beta 1) [24].

Several factors impact the phenotype and function of NK cells in liver disease, such as aging [25], calorie restriction [26], hepatitis viral infection [27], primary biliary cholangitis [28], HCC microenvironment [29], etc. In this review, we focus on the roles of NK cells in liver fibrosis, especially for the molecules that impact the interaction of NK cells with HSCs. The cellular and molecular mechanisms of the treatments that regulate NK cell function and phenotype in liver fibrosis are also reviewed and summarized.

## 2. The Phenotypes of NK Cells in the Liver

**Figure 1:**
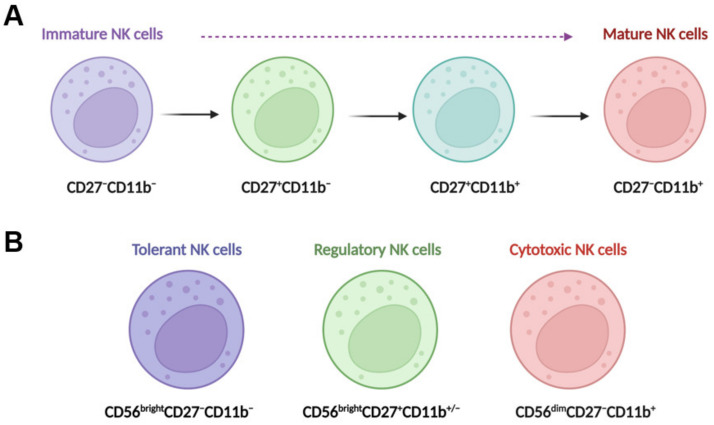
NK cell maturation and functional subsets. (**A**) Maturing NK cells can be divided into four populations by characterizing the expression of CD11b and CD27; these populations include CD11b^−^CD27^−^NK cells, CD11b^−^CD27^+^NK cells, CD11b^+^CD27^+^NK cells, and CD11b^+^CD27^−^NK cells. CD11b^−^CD27^−^NK cells are immature NK cells with differentiation potential, both CD11b^−^CD27^+^NK cells and CD11b^+^CD27^+^NK cells have the best ability to secrete cytokines, and CD11b^+^CD27^−^NK cells display high cytolytic function. (**B**) Human NK cells can also be classified into three functional subsets, tolerant NK cells (CD56^bright^CD27^−^CD11b^−^), regulatory NK cells (CD56^bright^CD27^+^CD11b^+/−^), and cytotoxic NK cells (CD56^dim^CD27^−^CD11b^+^).

In the liver, NK cells can be divided into two main populations: transient conventional NK (cNK) cells and liver resident NK (lr-NK) cells [15]. Mouse lr-NK cells are CD49a^+^DX5^−^NK cells, while their cNK cells are CD49a^−^DX5^+^NK cells [30]. The phenotype of “memory-like” NK (ml-NK) cells in mice is the same as the phenotype of lr-NK cells or CD49a^+^DX5^−^NK cells. Human cNK cells can be subdivided into CD56^dim^CD16^+^NK cells and CD56^bright^CD16^−^NK cells. In the human liver, CD56^dim^NK cells and CD56^bright^NK cells have similar frequencies [31]. Human liver CD56^bright^lr-NK cells are CCR5^+^CXCR6^+^CD69^+^NK cells, whereas CD56^dim^cNK cells lack expression of CD69, CCR5, and CXCR6 [32]. Human liver ml-NK cells are CXCR6^+^CD94/NKG2C^+^ (killer cell lectin-like receptor C2) NK cells [32]. The maturation of NK cells can be defined for four populations by characterizing the expression of CD11b and CD27 (Figure 1A), including CD11b^−^CD27^−^NK cells, CD11b^−^CD27^+^NK cells, CD11b^+^CD27^+^NK cells, and CD11b^+^CD27^−^NK cells [33,34]. CD11b^−^CD27^−^NK cells are immature NK cells with differentiation potential, both CD11b^−^CD27^+^NK cells and CD11b^+^CD27^+^NK cells have the best ability to secrete cytokines, and CD11b^+^CD27^−^NK cells display high cytolytic function [33]. Human NK cells can also be classified into three functional subsets (Figure 1B): tolerant NK cells (CD56^bright^CD27^−^CD11b^−^), regulatory NK cells (CD56^bright^CD27^+^CD11b^+/−^), and cytotoxic NK cells (CD56^dim^CD27^−^CD11b^+^) [35].

Change in NK phenotypes happens in liver disease. For example, peripheral activated CD56^bright^NK cells in patients with hepatitis B virus (HBV)-related decompensated liver cirrhosis (HBV-DLC) have been shown to have a phenotype with an increased expression of natural killer group 2, member D (NKG2D), perforin, and granzyme A/B and a decreased expression of inhibitory receptor CD158b1/2 compared NK cells from healthy controls, showing an immune activation status [36]. In contrast, the circulating CD56^dim^NK cells express low levels of CD107a and perforin with an impaired cytolytic capacity [36].

Single-cell RNA-sequencing (scRNA-seq) data illustrate that there are several subtypes of NK cells in chronic liver disease, which display multiple roles based on the different gene expressions. For example, scRNA-seq data analysis showed that the subpopulation of NK cells expressing *SELL*, a gene encoding the cell surface adhesion molecule L-selectin, was proliferated in alcohol-induced cirrhotic livers, whereas the subtype of NK cells expressing *XCL2* (X-C motif chemokine ligand 2) was enriched in the healthy livers [37]. The heterogeneity of peripheral NK cells has also been shown in the development of NASH from simple steatosis with a decreased expression of NK cell activation marker natural cytotoxicity receptor 3 (NCR3, also known as NKp30) in NASH [38]. In addition, several intracellular signaling mediators were less activated, including in the phosphorylation of extracellular signal-regulated protein kinases (pERK) and the phosphorylation of signal transducer and activator of transcription (pSTATs) 1, 2, 3, and 5 [38]. In the following sections, we will discuss the functions of NK cells in liver fibrosis and the important molecules involved in the cytotoxicity of NK cells against activated HSCs.

## 3. The Profibrotic Function of NK Cells

NK cells isolated from patients with chronic hepatitis C virus (HCV) infection and liver fibrosis (METAVIR fibrosis scores: F1 and F4) compared to NK cells isolated from healthy donors showed a significant reduction in the expression of NK activation markers CD107a (lysosomal-associated membrane protein 1, LAMP-1) and INF-γ [39]. In addition, these NK cells from HCV patients significantly promoted the proliferation of co-cultured human HSC cell line LX-2 cells, especially for the subpopulation that expressed intermediate intensity of α-smooth muscle actin (α-SMA) with small sizes according to the forward scatter profile in flow cytometry gating [39]. In addition, hepatic NKp44^+^NK cells from patients with HCV infection were positively correlated with liver fibrosis and viral load, producing TNF-α to promote liver injury [40]. In patients with HBV infection, transforming growth factor-beta (TGF-β) produced by activated HSCs can suppress the anti-fibrotic effect of NK cells by suppressing NK cell degranulation and IFN-γ production [41]. In patients with severe alcoholic hepatitis (SAH), NK cells can induce lysis of endothelial progenitor cells via the CX3CR1/fractalkine axis to promote inflammation and SAH progression [42]. Overall, in different liver disease conditions, NK cells have been demonstrated to be positively associated with the progression of liver fibrosis.

Recent studies showed that NK cells have long-term graft survival in patients with liver transplantation [43]. The recurrence of HCV infection in patients with liver transplantation can cause graft cirrhosis. The mismatch between the killer cell immunoglobulin-like receptor, two Ig domains, and long cytoplasmic tail 3 (KIR2DL3) and the ligands of human leukocyte antigen (HLA) class I antigens induces the progression of hepatitis to liver fibrosis [44]. Thus, inhibition of NK cell-mediated HSC activation in liver injury can prevent liver fibrosis.

## 4. The Anti-Fibrotic Function of NK Cells

**Figure 2:**
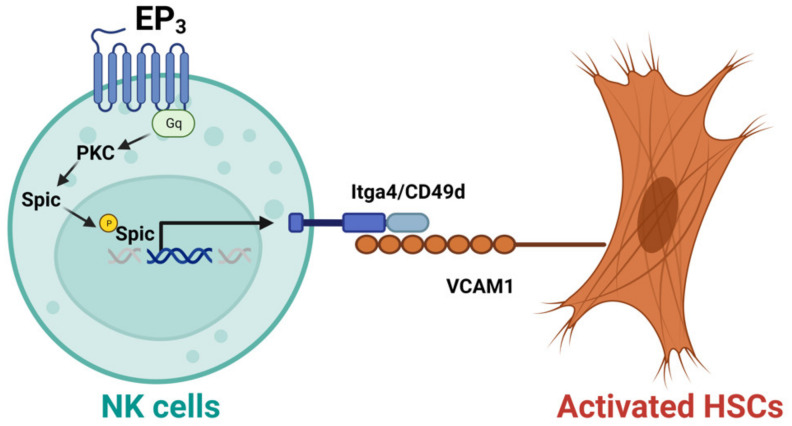
Activation of EP_3_ can improve the cytotoxicity of CD27^+^CD11b^+^ NK cells against activated hepatic stellate cells (HSCs). Mechanistically, activation of prostaglandin E receptor 3 (EP_3_) can increase the nuclear translocation of phosphorylated Spi-C transcription factor (Spic) by regulating protein kinase C (PKC) to upregulate integrin alpha-4 (Itga4, or CD49d) in NK cells. The binding of NK cells with activated HSCs is mediated by the interaction of Itga4 with vascular cell adhesion molecule 1 (VCAM1).

NK cells have also been shown to display anti-fibrotic effects in the liver. For example, CD27^+^CD11b^+^ NK cells can display cytotoxicity against activated HSCs via the interactions of integrin alpha-4 (Itga4, also known as CD49d)–vascular cell adhesion molecule 1 (VCAM1), which can be suppressed by depletion of prostaglandin E receptor 3 (EP_3_) [45]. Mechanistically, activation of EP_3_ can increase the nuclear translocation of phosphorylated Spi-C transcription factor (Spic) by regulating protein kinase C (PKC) to upregulate Itga4 expression in NK cells. The binding of NK cells and activated HSC cells is mediated by the interaction of Itga4 expressed by NK cells with VCAM1 expressed by activated HSCs [45], as shown in Figure 2. NK cells activated by natural product curcumin induced senescence of LX-2 cells and promoted fibrotic cell clearance by stimulating granule exocytosis [46]. The increased expression of NKG2D ligand major histocompatibility complex class I chain-related gene A (MICA) and UL16-binding protein 2 (ULBP2) on senescent LX-2 cells activate this process [46]. *MICA* is a polymorphic gene, and its alleles have been demonstrated to be associated with the histologic features of NASH such as liver inflammation and fibrosis [47]. However, the anti-fibrotic effect of NK cells can be suppressed by activated HSCs via producing TGF-β [48]. Therefore, understanding the molecular mechanisms that mediate the interaction of NK cells with activated HSCs or myofibroblasts can improve NK cell-mediated anti-fibrotic effects.

In addition, the anti-fibrotic effects of NK cells, as well as HSC activation, can be regulated by platelets. Platelets contain proteins and growth factors required for liver regeneration and repair [49], playing important roles in chronic liver diseases, including liver fibrosis [50,51]. For example, the platelet count can be used as a simple, non-invasive index to evaluate the degree of liver fibrosis in patients with CHB [52]. In addition, platelet-derived growth factor-β (PDGF-B) can activate HSCs to promote liver fibrosis [53]. PDGF-D not only enhances human NK cell effector functions by binding to the NKp44 receptor, it regulates IL-15-induced human NK cell survival by binding to PDGF receptor-beta [54]. Platelets also play an important role in NASH pathogenesis by promoting inflammatory cell accumulation, steatosis, and liver injury [55]. However, its function on NK cells remains to be further explored.

## 5. Cellular and Molecular Factors Interfere with the Interaction of NK Cells with HSCs

In chronic liver disease, liver resident and infiltrating cells can impact the function of NK cells and their cytotoxicity to HSCs. For example, mucosal-associated invariant T (MAIT) cells can activate the cytotoxicity of NK cells against HSCs in cholestatic mice with liver fibrosis induced by feeding a special diet (0.1%) or bile duct ligation (BDL) [56]. Several important molecules that are associated with the phenotype and function of NK cells are summarized. For example, CD96-expressing NK cells in human HCC tissues are functionally exhausted, with low expression levels of interleukin (IL)-15, IFN-γ, granzyme B, perforin, and TNF-α, but high levels of IL-10 and TGF-β1 [57]. Another study also showed that transcriptional factors such as *IRF8* (interferon regulatory factor 8), *NR4A2* (nuclear receptor subfamily 4 group A member 2), *IKZF3* (IKAROS family zinc finger 3), and *REL* (REL proto-oncogene, NF-κB subunit) may be implicated in the roles of NK cells during liver fibrosis [58]. In this section, we discuss the impact of liver cells and their secreting factors on the interaction of NK cells with HSCs.

### 5.1. CD96

CD96, an exhaustion marker for NK cells, can be induced by TGF-β1. Blockade of the interaction of CD96 and its ligand CD155 can restore the function of NK cells [57]. In addition, CD96 shares the ligand CD155 with both CD226 and T cell immunoreceptor with Ig and ITIM domains (TIGIT). CD226, also known as DNAM-1 (DNAX accessory molecule-1), can competitively bind with CD155 rather than CD96 to activate NK cell-mediated cytotoxic effects, whereas TIGIT controls the CD226-mediated effect on NK cells [59]. Multiparameter flow cytometry analysis showed that TIGIT expression was significantly increased and CD226 expression was suppressed in intrahepatic CD56^bright^NK cells compared to matched peripheral blood CD56^bright^NK cells [60]. Co-culture of peripheral blood CD56^bright^NK cells with human hepatoma cells or primary human hepatocyte organoids can increase the expression of TIGIT but suppress the expression of CD226 in NK cells, showing the phenotype of intrahepatic CD56^bright^NK cells [60]. Therefore, in the tumor microenvironment, the cytotoxicity of NK cells against cancer cells and activated HSCs can be suppressed due to the increased expression of CD96 and TIGIT.

### 5.2. CTLA-4

Regulatory T cells (Tregs) play a vital role in chronic liver diseases, including liver fibrosis and cancer [61]. Tregs can inhibit the activation of NK cells when co-cultured with HSCs in a cell-contact-dependent manner [62]. The cytotoxic T-lymphocyte antigen 4 (CTLA-4) expressed by Tregs can suppress the activation of NK cells by producing cytokines such as IL-8 and TGF-β1 to suppress the expression of NKG2D ligand MHC class I chain-related proteins A and B (MIC-A/B) and HLA class I on HSCs [62]. Therefore, reducing the number and function of Tregs or suppressing their expression of CTLA-4 may restore the cytotoxic function of NK cells against activated HSCs to prevent liver fibrosis.

### 5.3. EP_3_

The expression of EP_3_ was dramatically decreased in NK cells from mice with liver fibrosis and patients with liver cirrhosis [45]. Specific depletion of EP_3_ in NK cells can aggregate carbon tetrachloride (CCl_4_) or BDL–induced mouse liver fibrosis. The cytotoxicity of CD27^+^CD11b^+^NK cells against activated HSCs is suppressed by EP_3_ deletion [45]. In contrast, activating EP_3_ can increase the interaction of CD27^+^CD11b^+^NK cells and activate HSCs via Itga4-VCAM1 binding to promote NK cell cytotoxicity to ECM-producing HSCs [45]. Thus, EP_3_, as a G protein-coupled receptor (GPCR), can be targeted to increase NK cell function.

### 5.4. KIR

Killer-cell immunoglobulin-like receptor (KIR) is expressed on the surface of NK cells and regulates their cytotoxicity by interacting with HLA class I molecules [63]. The expression ratio of activating and inhibitory KIRs (a/iKIR) in NK cells changes in liver injury. Co-culture of lymphocytes from human patients with HCV infection can stimulate LX-2 cell activation and increase the expression of α-SMA compared with healthy lymphocytes. The α-SMA production in co-cultured cells was suppressed when HCV lymphocytes were treated with small interfering RNAs (siRNAs) against iKIR [64]. Adoptive transfer of splenocytes (enriched with NK cells) from naive SCID mice (lacking B and T cells) that underwent *iKIR* knockdown by siRNAs can reduce CCl_4_-induced liver fibrosis in BALB/c male SCID-Beige mice (lacking B/T/NK cells), which is evidenced by reduced expression of collagen and α-SMA production [64]. In addition, the interactions of KIR/HLA-I are associated with graft survival times of patients with liver transplantation, as well as NK cell alloreactivity [43].

### 5.5. KLRG1

The frequency of killer cell lectin-like receptor subfamily G member 1 (KLRG1)-expressing NK cells in the liver and blood of patients with CHB has been found to be negatively associated with liver fibrosis. Osteopontin derived from HSCs can stimulate the activation of NK cells to express CD44 and IFN-γ and increase NK cell degranulation activity [65]. In addition, KLRG1^+^ NK cells play an anti-fibrotic role in patients with CHB, which showed an increased production of IFN-γ and cytolytic activity against HSCs compared to KLRG1^−^ NK cells [65]. KLRG1 is a maturation marker of NK cells. Memory KLRG1^+^ NK cells post-HCV infection play an essential role in HCV clearance and mediate antigen-specific response against HCV infection [66].

### 5.6. Metabotropic Glutamate Receptor 5

Mice with NK cell-specific knockout metabotropic glutamate receptor 5 (mGluR5) had aggravated CCl_4_-induced liver fibrosis and decreased IFN-γ production compared to wild-type mice [67]. In contrast, activation of mGluR5 can increase NK cell cytotoxicity against activated HSCs by upregulating the expression of anti-fibrotic genes, such as *IFGN*, *PRF1* (perforin), and *KLRK1* (killer cell lectin-like receptor K1), and increasing IFN-γ production through the mitogen-activated protein kinase (MAPK)/extracellular signal-regulated kinase (ERK) pathway [67]. mGluR5 plays multiple roles in cell stress and metabolic regulation [68,69], making it a potential target for regulating NK cell function.

### 5.7. NKG2D Ligands or Stimulators

The expression of retinoic acid early inducible 1 (RAE-1), an NKG2D ligand, is undetectable in quiescent HSCs but highly expressed in activated HSCs [70]. Treatments such as polyinosinic-polycytidylic acid (poly(I:C)) or IFN-γ can increase the expression of NKG2D and TNF-related apoptosis-inducing ligands on liver NK cells to increase their cytotoxicity against activated HSCs [70]. Stimulated NK cells from patients with HCV or HIV (human immunodeficiency virus) with CD4^+^ T cell supernatants can enhance their cytotoxicity against HSCs compared to unstimulated NK cells. IL-2 secreted from CD4^+^ T cells contributes to the activation of NK cells and the upregulated expression of NKG2D in NK cells [71]. In addition, the frequency of circulating NK cells expressing NKG2D was also shown to decrease in patients with HCC [72]. IL-30 treatment has been shown to enhance the anti-fibrotic activity of NKT cells against activated HSCs by upregulating the expression of NKG2D and its binding with the ligand retinoic acid early inducible 1 (RAE-1) [73]. IL-30, a subunit of IL-27 (IL27p28), can also regulate both NK and T cell function by binding cytokine-like factor 1 [74]. Thus, IL-30 treatment may also enhance NK cell cytotoxicity against activated HSCs.

### 5.8. PD-1

The programmed death 1 (PD-1)/PD-ligand 1 (PD-L1) axis is involved in the pathogenesis of chronic liver diseases [75], such as hepatitis, liver fibrosis, and HCC. The frequency of NK cells was reduced in patients with HIV and HCV co-infection, which advanced the progression of liver fibrosis. In addition, an increased exhaustion marker PD-1 expression in NK cells was positively associated with advanced liver fibrosis and NK cell dysfunction [76]. Cancer-associated fibroblasts play a key role in liver cancer progression by inducing immune tolerance, chemoresistance, expression of growth factors, etc. [77]. Tumor cells can inhibit the function of NK cells via the interaction of PD-L1/PD-1 [78]. Therefore, inhibition of PD-1 expression in NK cells can improve their cytotoxicity against cancer and fibrotic cells.

### 5.9. Siglecs

Sialic acid-binding immunoglobulin-like lectins (Siglecs) play an important role in the regulation of immune cell functions [79,80], including in NK cells [81]. Siglec-7 and Siglec-9 are highly expressed by NK cells. In patients with NAFLD and different stages of liver fibrosis, the frequency of peripheral blood Siglec-7^+^CD56^dim^NK cells was suppressed, whereas the frequency of dysfunctional Siglec-7^−^PD-1^+^CD57^+^CD56^dim^NK cells was increased [11]. The expression of Siglec-9 on NK cells in patients with CHB was decreased, which was negatively correlated with serum hepatitis B e antigen (HBeAg) and HBV DNA titer [8]. Therefore, regulating Siglec expression in NK cells can improve their function.

### 5.10. STATs

The signal transducer and activator of transcription 1 (STAT1) is a transcription factor that regulates NK cell proliferation and activation. NK cell dysfunction is mediated by STAT1 by downregulating NKG2D expression. Cellular studies show that STAT1 deletion in human NK cell line NK-92 cells can inhibit cell proliferation, promote cell apoptosis, and impair their cytotoxicity [27]. Treatment of dihydromyricetin (DHM) can inhibit HSC activation in vitro and decrease CCl_4_-induced liver fibrosis in C57BL/6 mice. Further molecular studies show that DHM treatment can improve NK cell-killing ability by enhancing IFN-γ expression through the NF-κB/STAT3 pathway [82]. Therefore, regulation of the expression of transcription factor STAT may be applied to regulate NK cell function.

### 5.11. TIGIT

In addition to the suppressive effect of TIGIT on CD226-mediated activation of NK cells [59], TIGIT deficiency can suppress parasite (*Schistosoma japonicum*) infection-induced liver fibrosis [83]. A cellular mechanism study showed that NK cells from TIGIT-deficient mice compared to NK cells from wild-type mice can induce more co-cultured HSC apoptosis [83].

Overall, these molecules (Table 1) contribute to the regulation of the cytotoxicity of NK cells against HSCs. Regulation of these genes could provide therapeutic strategies against liver fibrosis.

**Table 1:** Molecules regulate the interaction of NK cells with HSCs.

Molecules	Function	References
CD96	CD96, an exhaustion marker for NK cells, can be induced by TGF-β1. Blockade of the interaction of CD96 and its ligand CD155 can restore the function of NK cells.	[57]
CTLA-4	CTLA-4 expressed by Tregs can suppress NK cell activation by releasing cytokines such as IL-8 and TGF-β1 to suppress the expression of NKG2D ligand MHC class I chain-related proteins A and B (MIC-A/B) and HLA class I on HSCs.	[62]
EP3	Specific depletion of E-prostanoid 3 receptor (EP_3_) in NK cells can aggregate carbon tetrachloride (CCl_4_)- or bile duct ligation (BDL)-induced mouse liver fibrosis. The cytotoxicity of CD27^+^CD11b^+^NK cells against activated HSCs is suppressed by EP3 deletion.	[45]
KIR	Adoptive transfer of splenocytes enriched with NK cells that have undergone inhibitory killer-cell immunoglobulin-like receptor (iKIR) knockdown by small interfering RNAs (siRNAs) can reduce CCl_4_-induced liver fibrosis in BALB/c male SCID-Beige mice (lacking B/T/NK cells), evidenced by reduced expression of collagen and α-SMA.	[64]
KLRG1	The frequency of killer cell lectin-like receptor subfamily G member 1 (KLRG1)-expressing NK cells displayed anti-fibrotic effects in patients with chronic hepatitis B (CHB).	[65]
mGluR5	Mice with NK cell-specific knockout metabotropic glutamate receptor 5 (mGluR5) have aggravated CCl_4_-induced liver fibrosis compared to wild-type mice. In contrast, activation of mGluR5 can increase NK cell cytotoxicity against activated HSCs by upregulating the expression of anti-fibrotic genes, such as *PRF1* (perforin), *KLRK1* (killer cell lectin-like receptor K1), and IFN-γ production.	[67]
NKG2D	Treatments such as poly(I:C) or IFN-γ can stimulate the expression of NKG2D on NK cells to increase its cytotoxicity against activated HSCs.	[70]
PD-1	The expression of exhaustion marker PD-1 in NK cells was positively associated with advanced liver fibrosis and NK cell dysfunction.	[76]
Siglec-7	The frequency of peripheral blood Siglec-7^+^CD56^dim^NK cells was decreased in patients with NAFLD with different stages of liver fibrosis, whereas the frequency of dysfunctional Siglec-7^−^PD-1^+^CD57^+^CD56^dim^NK cells was increased.	[11]
STAT1	STAT1 deletion in human NK cell line NK-92 cells can inhibit cell proliferation, promote cell apoptosis, and impair cell cytotoxicity.	[27]
STAT3	Treatment of dihydromyricetin (DHM) can inhibit HSC activation in vitro and decrease CCl_4_-induced liver fibrosis in C57BL/6 mice by improving NK cell killing ability and IFN-γ expression through the NF-κB/STAT3 pathway.	[82]
TIGIT	TIGIT deficiency can inhibit parasite (*Schistosoma japonicum*) infection-induced liver fibrosis by increasing NK cell-mediated apoptosis HSCs.	[83]

Abbreviations: α-SMA: α-smooth muscle actin; CTLA-4: cytotoxic T-lymphocyte antigen 4; HSCs: hepatic stellate cells; NF-κB: nuclear factor-κB; NKG2D: natural killer group 2, member D; Siglec-7: sialic acid-binding immunoglobulin-like lectin 7; Poly(I:C): polyinosinic-polycytidylic acid; Tregs: regulatory T cells; STAT1/3: signal transducer and activator of transcription 1/3; TIGIT: T cell immunoreceptor with Ig and ITIM domains.

## 6. Treatments Improve the Cytolytic Function of NK Cells to HSCs

Factors such as pathogenic infection (e.g., infection of *Echinococcus multilocularis*), liver metabolic disorder (e.g., NAFLD), and tumor development can induce NK cell dysfunction, leading to liver fibrosis [11,84,85]. Some treatments have been shown to regulate NK cell function to inhibit liver fibrosis, such as alcohol dehydrogenase 3 (ADH3) inhibitors, microRNAs, NKG2D activators, and natural products. For example, treatment with cultured mycelium of *Cordyceps sinensis* can significantly inhibit CCl_4_ treatment-induced NK cell reduction and dysfunction in mice with liver fibrosis [86]. Here, we review some treatments that regulate NK cell function against fibrosis.

### 6.1. ADH Inhibitor

The activity of alcohol dehydrogenase (ADH) has been shown to be positively associated with liver fibrosis in mice, as well as the expression of HSC activation markers collagen (Col)1a1 and α-SMA [87]. Treatment with 4-methylpyrazole (4-MP), a broad inhibitor ADH, can attenuate CCl_4_- and BDL-induced liver fibrosis in mice by regulating IFN-γ production in NK cells to increase the apoptosis of activated HSCs [88]. Meanwhile, HSCs isolated from ADH3-deficient mice expressed lower levels of collagen and TGF-β1 compared with those in wild-type mice. In contrast, the expression of IFN-γ was increased in NK cells from ADH3-deficient mice compared to that in NK cells from wild-type mice [89].

### 6.2. MicroRNAs

Exosomes derived from NK cells can reduce TGF-β1-induced HSC activation. A molecular mechanism study demonstrates that microRNA-233 (miR-233) expressed in NK cell exosomes can block autophagy during HSC activation by inhibiting autophagy-related 7 (ATG7) expression [90]. Similarly, one study shows that miR-96-5p can block the autophagy activity of LX-2 cells by targeting ATG7 to suppress the mRNA expression levels of ECM genes [91]. Another study also shows that miR-155 can upregulate the expression of IFN-γ in NK cells from patients with HCV by suppressing the expression of T cell immunoglobulin- and mucin-domain-containing molecule 3 (Tim-3) [92]. Thus, microRNAs can be applied to improve NK cell cytotoxicity against activated HSCs and directly inhibit HSC activation.

### 6.3. NKG2D Stimulators

The expression of NK cell activating ligand RAE-1, a ligand for NKG2D receptor, was upregulated on early activated HSCs but lost in fully activated HSCs. Lacking RAE-1 expression makes fully activated HSCs resistant to NK cell cytotoxicity [93]. In mice with primary biliary cholangitis (PBC), NK cells can be activated by Kupffer cells, a process mediated by NKG2D/RAE-1 interaction to produce IFN-γ synergistically with Kupffer cell-derived TNF-α, resulting in liver inflammation [94]. In patients with HIV and HCV co-infection, CD4^+^ T cells can stimulate the anti-fibrotic activity of NK cells via IL-2-induced upregulation of NKG2D [71]. Overall, NKG2D can be targeted to induce NK cell activation and increase their anti-fibrotic activity.

### 6.4. NLRP3 Inflammasome Regulation

The NLRP3 (NOD-, LRR-, and pyrin domain-containing protein 3) inflammasome is comprised of a sensor (NLRP3), an adaptor (apoptosis-associated speck-like protein containing a caspase recruitment domain/ACS), and an effector (caspase 1) [95]. NLRP3 is an intracellular sensor that detects a wide range of microbial- and host-derived signals [96]. NLRP3 ablation in HCC cells can upregulate their expression of MIC-A, which interacts with NKG2D in NK cells to enhance NK cytotoxicity. In addition, mice xenografted with NLRP3-knockout HCC cells developed tumors slowly, and tumor cells were sensitive to NK cell cytotoxicity [97]. In contrast, the NLRP3 inflammasome-IL18 signaling pathway in Kupffer cells can prime hepatic NK cell maturation and anti-cancer activity [98]. The function of NLRP3 inflammasome on NK cell activation is cell-dependent.

### 6.5. Ras Homology Family Member A (RhoA) Kinase Inhibitor

The Ras homolog gene family member A (RhoA) is a Rho GTPase superfamily member, and it plays a vital role in signal transduction and regulation of gene transcription. Therefore, RhoA is involved in many cellular functions, such as cell division, proliferation, and migration [99]. Treatment with Fasudil, a RhoA kinase inhibitor, can ameliorate thioacetamide (TAA)-induced liver fibrosis in mice by stimulating NK cell activation and suppressing HSC activation and proliferation [100]. RhoA is also involved in the PDGF-BB (platelet-derived growth factor-BB) or TGF-β1-induced activation of HSCs, which can be suppressed by anti-fibrotic treatment [101]. Therefore, RhoA is a potential target for the treatment of liver fibrosis.

### 6.6. γδT Cells

In addition to their direct killing ability against activated HSCs, γδT cells can increase the cytotoxicity of cNK and lr-NK cells against activated HSCs to improve liver fibrosis. A molecular study further shows that the crosstalk between γδT and NK cells is mediated partly by CD137, the co-stimulatory receptor 4-1BB [102].

### 6.7. Tim-3

The expression of Tim-3 in circulating NK cells and liver infiltrating lymphocytes in patients with CHB was significantly increased compared to that in healthy controls [103]. Upregulation of Tim-3 suppressed NK cell cytotoxicity and IFN-γ production [103]. Rosuvastatin, a lipid-lowering agent, can significantly reduce the percentage of Tim-3^+^ cells in NK cells of patients with CHB compared with the placebo group [104]. A higher frequency of NK cells co-expressing Tim-3 and TIGIT was found in patients with chronic HCV with advanced fibrosis, with increased susceptibility to HCC development [105]. As an immune checkpoint receptor, Tim-3 can suppress the effects of NK cells, which may be targeted to improve NK cell cytotoxicity to activated HSCs.

## 7. Liver Resident NK Cells

**Figure 3:**
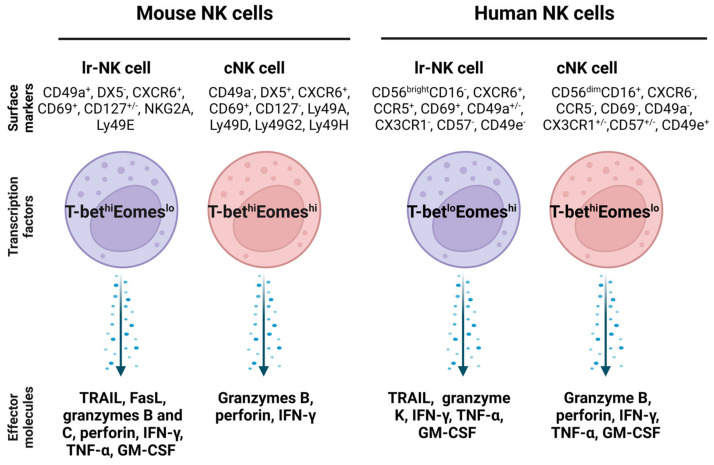
Comparison of mouse and human liver resident NK cells and conventional NK cells. The transcription factors in lr-NK cells and cNK cells are different, and they also express different surface markers and effector molecules. Abbreviations: cNK: conventional NK cell; Eomes: eomesodermin; FasL: Fas ligand; GM-CSF: granulocyte-macrophage colony-stimulating factor; lr-NK: liver resident NK; NKG2A: killer cell lectin-like receptor C1; T-bet: T-box transcription factor; TRAIL: TNF-related apoptosis-inducing ligand.

In the human liver, NK cells account for about 50% of intrahepatic lymphocytes and play important roles in liver immune responses [15]. Lr-NK cells (liver-resident NK cells) are a heterogeneous population and exhibit phenotypical differences from cNK (conventional NK) cells [106]. The features of lr-NK and cNK cells in mice and humans are summarized [106], as shown in Figure 3.

Lr-NK cells have been shown to have distinct gene expression profiles and display important roles in autoimmune cholangitis [107] and liver fibrosis [108]. One study shows that the frequency of intrahepatic NKp44^+^ NK cells producing TNF-α is correlated with both HCV infection level and stage of liver fibrosis [40]. Another study reveals that human lr-NK (CD49a^+^CD25^+^) cells expressing CD25, CD34, and CXCR3 display high proliferative capacity in vitro [109]. In contrast, recruited CXCR3^+^ NK cells in the livers of mice with diet-induced NASH display a protective role against liver fibrosis. However, the distinct roles of both lr-NK and cNK cells in the pathogenesis of liver fibrosis remain to be explored.

Accumulating studies reveal that targeting lr-NK cells can prevent liver fibrosis and cancer progression. For example, treatment with *Ecballium elateriumone*, a medicinal plant, increased the number of lr-NK cells and their expression of CD107a and IFN-γ and inhibited thioacetamide-induced liver fibrosis in mice [108]. Treatment with low doses of IL-2 can increase the proliferation of CD49a^+^CD25^+^lr-NK cells in cirrhotic livers [109]. γδT cell subset (γδT1) cells expressing IFN-γ directly display cytotoxicity against activated HSCs, which can also enhance the cytotoxicity of lr-NK cells against activated HSCs [102]. Some molecules can be targeted to enhance the activity of lr-NK cells. One study showed that lr-NK cells expressed TNF-related apoptosis-inducing ligand (TRAIL), distinguishing them from cNK cells. Treatment with Everolimus, a mammalian target of rapamycin (mTOR) inhibitor, can improve the anti-tumor activity of lr-NK cells by upregulating TRAIL expression [110]. Another study shows that retinoid-related orphan nuclear receptor alpha (RORα) is highly expressed in lr-NK cells, and its agonist can activate lr-NK cells to prevent colorectal cancer liver metastasis [111]. In addition, chemokine receptors such as CXCR6 and CCR5 (Figure 3) are also only expressed by human lr-NK cells, but not human cNK cells, which could be targeted to modulate lr-NK cell function to inhibit liver fibrosis [32]. For example, treatment with cytokines such as IL-12 and IL-15 can increase the expression of CXCR6 and CD49a in circulating NK cells to display comparable phenotypic and functional features of lr-NK cells [112]. Furthermore, some factors such as aryl hydrocarbon receptors are required for the maintenance of lr-NK cells [113], and can be targeted for regulating lr-NK cell functions. Overall, targeting molecules such as TRAIL, RORα, and chemokine receptors can increase the cytotoxicity of lr-NK cells against cancer cells and activated HSCs.

## 8. Clinical Evaluations

In clinical trials, some treatments have been evaluated to treat liver diseases by regulating NK cell frequency and function, including lipid-lowing agents (e.g., rosuvastatin) [104], immune regulators (e.g., Toll-like receptor 8 agonist GS-9688) [114,115], antiviral agents (e.g., pegylated interferon-alpha) [116,117,118], anticancer agents (e.g., Sorafenib) [119], microRNAs (e.g., RG-101) [120], and combined therapies (e.g., IL-2 and IFN) [121], as well as organisms (e.g., *Arthrospira*, a genus of free-floating filamentous cyanobacteria) [122]. In Table 2, we review the functions of different treatments in NK cell regulation. However, studies that target NK cells for liver fibrosis treatment are rare, and more investigations are expected.

**Table 2:** Treatments regulate NK cells.

Treatment	Function	References
Rosuvastatin (RSV)	RSV administration can significantly increase CD3^+^ CD16^+^ CD56^+^ NKT cells and reduce the percentage of Tim-3^+^NK cells in patients with CHB compared to the controls.	[104]
TLR-8 agonist GS-9688	In vitro treatment with GS-9688 can induce the expression of interferon-γ and TNF-α in NK cells to enhance their cytolytic function against hepatocytes. In addition, GS-9688 can increase the frequency of activated NK cells in patients with HBV.	[114]
TLR 7 agonist GS-9620	Administration of GS-9620 increased NK cell activation and function but did not decrease the levels of HBsAg in patients with suppression of HBV infection by nucleos(t)ide analogue (NA) therapy.	[115]
A combination of pegylated interferon-alpha (peg-IFN-α) and NA therapy	The combined treatment can effectively reduce HBsAg by increasing the frequency and absolute number of circulating CD56^bright^NK cells, compared with the NA treatment group, whereas the CD56^dim^NK cells were decreased.	[116]
Peg-IFN-α and sequential NA treatment	CHB patients receiving Peg-IFN-α and sequential NA treatment showed a decrease in HBsAg due to an increase in a subset of distinct NK cells, expressing NK cell activation receptors NKp30 and NKp46 with increased IFN-γ production and cytotoxicity.	[117]
Peg-IFN-α-2b monotherapy or combination therapy with adefovir dipivoxil	The frequency and the absolute number of NKp30^+^NK cells were significantly increased, which was accompanied by increased expression of CD107a and IFN-γ in patients with CHB during Peg-IFN-α-2b monotherapy or combination therapy with adefovir dipivoxil.	[118]
Sorafenib	Sorafenib treatment can reduce the percentage of CD56^bright^CD16^−^NK cells and increase the frequency of CD56^dim^CD16^+^NK cells and the expression of granzyme B and perforin in total NK cells and both subsets of cells, improving the overall survival in HCC patients.	[119]
RG-101, an N-acetylgalactosamine-conjugated anti-microRNA-122 oligonucleotide	A single subcutaneous administration of RG-101 (2 mg/kg) increased the frequency of NK cells in PBMCs and decreased plasma HCV RNA and IFN-γ-induced protein 10 (IP-10) levels in patients.	[120]
IFN or IL-2 and therapeutic vaccine with IFN	Combination therapy with IFN and other immunomodulators such as IL-2 can enhance HBeAg in patients with entecavir treatment by significantly increasing peripheral CD56^bright^CD16^−^NK cells and decreasing regulatory T cells.	[121]
Arthrospira	Treatment with *Arthrospira*, a genus of free-floating filamentous cyanobacteria, increased serum IFN-γ levels but decreased serum TNF-α and IL-6 and hepatic fibrosis and steatosis in CHB patients receiving NA therapy.	[122]

Abbreviations: CHB: chronic hepatitis B (CHB); HBeAg: hepatitis B e antigen; HBsAg: hepatitis B surface antigen; IFN: interferon; PBMCs: peripheral blood mononuclear cells; TLR-8: Toll-like receptor 8.

Chimeric antigen receptor (CAR) engineered NK cells are potent strategies for the treatment of liver cancer and fibrosis in the new decade due to their specificity and lower side effects [123]. Several recruiting clinical trials aim to study the efficacy of NK cells in treatments against HCC and hepatitis viral infection (ClinicalTrials.gov.; e.g., NCT05040438, NCT04162158, NCT05171309, and NCT03761875, accession date: 28 March 2023).

In the future, combined therapies will advance the efficacy and safety of NK cell-mediated treatment. One clinical trial reveals that a combined therapy of short-term irreversible electroporation and allogenic NK cell immunotherapy significantly reduces circulating tumor cells and increases immune function in patients with unresectable primary liver cancer [124]. Another clinical trial shows that the combination of locoregional high-dose autologous NK cell therapy with hepatic arterial infusion chemotherapy is a safe and effective therapy in patients with locally advanced HCC who are refractory to the standard treatment [125]. However, the development of NK cells targeting activated HSCs to treat liver fibrosis is less investigated.

## 9. Summary

Uncontrolled progression of liver fibrosis advances the development and progression of liver cirrhosis and HCC. However, there are no currently available FDA-approved anti-fibrotic treatments, even though there has been great progress in understanding the pathogenesis of liver fibrosis. Activated HSCs and differentiated myofibroblasts contribute to liver fibrosis through excessive ECM production. Cellular crosstalk between HSCs and surrounding cells, including both liver parenchymal and non-parenchymal cells, is involved in the activation of HSCs. Among them, NK cells play a crucial role in combating liver fibrosis and its causative factors, such as viral infections. Regulating the role of NK cells can suppress the activation of HSCs and improve their cytolytic function against activated HSCs or myofibroblasts to reverse liver fibrosis. Cells such as Tregs and molecules such as EP_3_ can regulate the cytotoxic function of NK cells. In addition, treatments such as ADH3 inhibitors, microRNAs, NKG2D activators, and natural products have been shown to regulate NK cell function to inhibit liver fibrosis. To date, the only available treatments, such as lipid-lowing agents, immune regulators, antiviral and anti-cancer agents, microRNAs, and combined therapies (e.g., IL-2 and IFN), regulate NK cell activation to inhibit fibrosis-causing factors. Therapeutic NK cells (e.g., CAR NK cells) or NK cell function regulators are limited. Overall, a better understanding of the cellular and molecular factors that interfere with the interaction between NK cells (especially lr-NK cells) and HSCs will provide new therapeutic targets for liver fibrosis. Meanwhile, more clinical trials are expected to investigate the roles of NK cells and NK cell-mediated factors in liver fibrosis treatment. 

## Data Availability

All data supporting reported results can be found in this paper.
